# Supplementary material for: Influence of exercise commitment on exercise adherence among undergraduate students: Chain mediation role of health beliefs and exercise behavior
Source: PLoS One. 2025 Nov 18;20(11):e0337226. doi: 10.1371/journal.pone.0337226 (PMC12626266; doi:10.1371/journal.pone.0337226)
Supplement: Attachment — Submitted filename: Response to Reviewers.docx [file pone.0337226.s001.docx]

**Comments from the Academic Editor:**

1. The article format was revised in accordance with the formatting requirements of PLOS ONE.

2. The funding disclosure was modified and stated in the Cover Letter.

3. The raw data of the current manuscript was deposited in a public resource repository, with open and unrestricted access.

4. The raw data has already been uploaded to the public resource repository.

5. The titles of the supporting information files were revised.

6. The referenced works were updated.

**Comments from the Reviewers:**

1. Reviewer 1’s first comment pointed out that there were significant discrepancies in the funding disclosure. The financial disclosure has now been stated in the Cover Letter. The correct financial disclosure is as follows: Guangxi Science and Technology Normal University High-Level Talent Research Start-Up Project (GXKS2024GKY006).

2. Reviewer 1’s second comment indicated inconsistency regarding data availability. The raw data of the manuscript was already deposited in a public resource repository, with open and unrestricted access. The uploaded data fully supports all research results in this study. Data access link: <https://doi.org/10.6084/m9.figshare.29604041。>

3. Reviewer 1’s third comment indicated that Reference 5 was a news article and an online early release, which does not meet publication requirements. We replaced it with a 2023 research article.

4. Reviewer 1’s fourth comment indicated that the authors acknowledged the limitations of the cross-sectional design but did not sufficiently discuss its impacts. The discussion was revised to more clearly highlight the shortcomings of the cross-sectional study and propose improvement strategies.

5. Reviewer 1’s fifth comment indicated that lines 235–236 stated, "Exercise commitment significantly and positively predicts health beliefs (β = 0.591, p < 0.01), which does not support Hypothesis 1." This has now been revised to: "Exercise commitment significantly and positively predicts health beliefs (β = 0.591, p < 0.01), which supports the first path in the mediation model."

6. Reviewer 1’s sixth comment indicated that the practical implications in the discussion section were unclear (e.g, “optimizing the exercise environment”). Based on the research results, more specific and feasible suggestions have now been provided.

7. Reviewer 2 indicated inconsistencies in the funding statement. The mistake in the funding section have now been clarified and corrected.

8. Reviewer 3 indicated inconsistencies regarding data availability. The accessible data has now been uploaded to the repository.

9. Reviewer 4 suggested making it clear what preventive measures were implemented to reduce such biases. The preventive measures taken to minimize common method bias have now been explicitly explained.

10. Reviewer 4 indicated that the limitations of the cross-sectional study and proposed solutions should be clearly stated. We further described the limitations of the cross-sectional study and proposed future longitudinal and experimental research in the future study.

11. Reviewer 4 requested that the dataset should be uploaded to a public repository. The raw data was already uploaded to the public resource repository.
