# Supplementary material for: Influence of exercise commitment on exercise adherence among undergraduate students: Chain mediation role of health beliefs and exercise behavior
Source: PLoS One. 2025 Nov 18;20(11):e0337226. doi: 10.1371/journal.pone.0337226 (PMC12626266; doi:10.1371/journal.pone.0337226)
Supplement: Attachment — Submitted filename: Response to Reviewers.doc [file pone.0337226.s002.doc]

****Dear reviewer:****

**Thank you very much for your meticulous review. Here are my answers to your questions and the relevant revisions to the article. The modified parts are marked in red in the article.**

****Response to Academic Editor****

1.If the reviewer comments include a recommendation to cite specific previously published works, please review and evaluate these publications to determine whether they are relevant and should be cited. There is no requirement to cite these works unless the editor has indicated otherwise. Please review your reference list to ensure that it is complete and correct. If you have cited papers that have been retracted, please include the rationale for doing so in the manuscript text, or remove these references and replace them with relevant current references. Any changes to the reference list should be mentioned in the rebuttal letter that accompanies your revised manuscript. If you need to cite a retracted article, indicate the article’s retracted status in the References list and also include a citation and full reference for the retraction notice.

**Response:**We have made modifications and marked them in red in the reference.

****Response to Additional Editor Comments****

1.The sample is restricted to universities in Guangxi Province, which may limit external validity. This should be explicitly stated in both the Abstract and Conclusion to avoid overgeneralization to all undergraduates.

**Response:** We have added relevant content.

“However, as the sample was drawn exclusively from university students in Guangxi Province, the generalizability of the findings is limited. Future studies should expand the sample scope to include a more diverse population”**Line 30-32.**“However, since the study merely incorporated university students from Guangxi Province, the generalizability of the findings is constrained”**Line 386-387.**

2.(Exercise Behavior vs. Exercise Adherence):While measured with different instruments, the conceptual distinction between these constructs could be further clarified in the Methods.

**Response:** We have added relevant content.

“Exercise behavior refers to an individual's engagement in physical activities. Exercise adherence denotes the ability and willingness to maintain such behavior over an extended period. In essence, it represents the sustained manifestation of exercise behavior” **Line 178-181.**

3.The manuscript applies both Social Cognitive Theory and the Health Belief Model but largely in parallel. Greater emphasis on how their integration offers novel explanatory power would strengthen the contribution.

**Response:** We have added relevant content.

“The effective integration of the two provides a more comprehensive explanation of the cognitive-behavioral mechanisms underlying individual actions. The establishment of exercise commitment reflects an individual's stronger pursuit of and belief in health, while health belief, in turn, effectively promotes positive social-cognitive processes that reinforce exercise behavior” **Line 84-88.**

4.Recommendations such as “incentive mechanisms” remain vague. Expanding this section with concrete examples (e.g., digital self-monitoring tools, peer-led initiatives, or curriculum-based interventions) would enhance applied value.

**Response:** We have added relevant content.

“For example, implementing diversified sports programs, using digital self-monitoring tools to identify and reward students who maintain regular exercise, establishing a campus competition system with weekly sporting events, incorporating exercise duration into course assessments, and continuously providing positive reinforcement along with moderately challenging physical tasks can all enhance students' enjoyment of exercise”**Line 296-301.**

5.Some foundational citations are dated (e.g., Glanz, 1997). Consider balancing with more recent literature (2022–2024) to reflect current advances. Read and cite https://doi.org/10.1186/s40359-025-02767-0 ;https://doi.org/10.1108/QEA-06-2024-0054.

**Response:**We have made modifications and marked them in red in the reference.

****Response to Reviewers****

1.There is a significant discrepancy concerning the ethics committee approval number that must be resolved prior to publication. This is a critical issue for research integrity and reproducibility.Inconsistent Ethics Approval Numbers: In the "Ethics Statement" section on page 4, the approval number is listed as GKSTY-2024032. However, in the main body of the text, under section "2.1 Survey Subject" on page 15, the approval number is cited as GKSTY-2023056.

**Response:**We have made modifications.

“(Approval No: GKSTY-2024032)”**Line173.**
